# Evaluation of a Tool to Measure Pharmacists’ Readiness to Manage Intimate Partner Violence

**DOI:** 10.3390/pharmacy6030066

**Published:** 2018-07-12

**Authors:** Marie Barnard, Donna West-Strum, Yi Yang, Erin Holmes

**Affiliations:** Department of Pharmacy Administration, School of Pharmacy, University of Mississippi, 223 Faser Hall, University, MS 38677, USA; dswest@olemiss.edu (D.W.-S.); yiyang@olemiss.edu (Y.Y.); erholmes@olemiss.edu (E.H.)

**Keywords:** community pharmacy, intimate partner violence, advanced pharmacy services, assessment, continuing professional education, pharmacy education

## Abstract

Intimate partner violence (IPV) is a public health problem that demands a comprehensive health care response. Provider education and training is needed for the entire health care team, including pharmacists, to competently care for IPV-impacted patients. Standardized assessments are needed to determine need for training and to evaluate the effectiveness of IPV training initiatives. The Physician Readiness to Manage Intimate Partner Violence Survey (PREMIS) has previously been validated for physicians. This study adapted and evaluated the PREMIS instrument for use with pharmacists to assess knowledge, attitudes, behaviors, and intentions related to IPV and IPV screening. A total of 144 surveys from community pharmacists were analyzed. Pharmacists had low levels of IPV knowledge. Exploratory factor analysis revealed a five-factor structure: workplace and self-efficacy, preparation, legal requirements, alcohol and drugs, and constraints. This instrument can be utilized to guide the development and implementation of IPV-related training initiatives for pharmacists.

## 1. Introduction

Intimate partner violence (IPV) is a public health problem of epidemic proportion in the United States, impacting more than 11 million people each year [1]. IPV has negative health impacts that include physical injuries, exacerbation of chronic medical conditions, harmful mental health impacts, and poor health behaviors, including poor medication adherence [2,3,4,5,6,7]. The health care system has recognized the seriousness of IPV and has been actively recommending screening for over two decades [8,9,10]. While the guidance and standards of care for most health care providers call for routine screening, and universal screening is widely promoted, most investigations have found that screening is poorly adopted and implemented in practice [4,11,12,13,14,15,16]. Clearly, there is a need to expand the health care response to IPV.

One health care field, pharmacy, has not been included in the effort to address IPV. This is unfortunate because for many patients, pharmacists are the health care professional with whom they have the most accessible and frequent contact. Engaging community pharmacists in this public health effort could provide an additional opportunity to improve the care of IPV-impacted patients. Pharmacists are actively involved in public health initiatives, from counseling patients on smoking cessation and diabetes management, to health screenings and provision of vaccinations [17,18,19,20,21]. Patients report that pharmacists are one of the most trusted members of the health care team and utilization of the expanding pharmacy services continues to increase, including engagement in partnerships in public health [22,23,24,25,26]. Patients exposed to IPV are more likely to utilize prescription medications, including mental health and antihypertensive drugs [27,28,29]. Patients exposed to IPV are at greater risk for cost-related medication nonadherence and are less likely to utilize contraception [30,31]. Given that IPV negatively impacts health behaviors, including medication compliance, increased knowledge and awareness of IPV and its impact on patients can improve pharmacy care and provide an additional prevention and intervention opportunity within the health care system to address IPV [6,32,33].

The development of education and training programs to prepare pharmacists to care for IPV-impacted patients requires assessment tools to evaluate training outcomes. Currently, no such instrument exists for use with pharmacists. It is difficult to develop successful educational and training programs to build skills and improve confidence in treating these patients without a tool to better understand health care providers’ educational needs and to assess training program outcomes. One such instrument has been developed for measuring physicians’ readiness to manage IPV. The instrument, developed and validated by Short et al. in 2006, is the PREMIS (Physician Readiness to Manage Intimate Partner Violence Survey) [34]. PREMIS is a 67-item comprehensive survey that measures a physician’s preparedness to manage IPV patients. The tool examines knowledge, attitudes, beliefs, and self-reported practice behaviors related to IPV. The survey items were developed by a review of existing survey tools in the literature. A content analysis was conducted through review by an outside group of IPV educators. The characteristics of the instrument were evaluated in two separate populations of physicians. PREMIS was shown to be reliable and valid, sensitive to change, and capable of discriminating trained from untrained providers [34]. Construct validity checks included evaluation of the Rand coefficient for the relationship between the empirically derived scales and the objective values assigned to the original theoretical constructs developed by the expert panel. The Rand coefficient was 0.89, indicating a high degree of association between the original theoretical constructs and the empirically derived scales. Construct validity was also assessed by examining the correlations between related instrument scales and the extent to which self-evaluated knowledge, attitudes, and beliefs predicted self-reported behaviors. These analyses found significant correlation between scores on practice issues, all background scales, actual knowledge, and six of the eight opinion scales (alcohol/drugs and victim autonomy were not significantly associated with practice issues). An external validity study conducted site visits to physicians’ offices and compared observed practice activities to reported practice activities related to IPV and found a high correlation between the two.

Two other studies have examined the PREMIS instrument. The first adapted it for use in a population of students in medicine, nursing, social work, and dentistry [35]. A factor analysis of the adapted student PREMIS instrument identified six of the eight factors identified in the original PREMIS instrument (see Table 1). The Workplace Issues and the Constraints scales were not identified, which was expected as this was a student population. The adapted instrument found a new scale, IPV screening, that had good reliability (α = 0.74). The Connor et al. study demonstrated that the PREMIS scale can successfully be modified for use in other provider groups in addition to physicians. The second study translated the PREMIS instrument into another language (Greek) and tested it in a sample of primary care physicians in Greece [36]. The translated measure found all of the scales found in the original PREMIS study and the IPV screening scale found in the student study (see Table 1). The PREMIS instrument has demonstrated that it can be adapted for other health care provider groups and settings, making it an ideal scale for adapting to the pharmacist population. The development of a pharmacy-specific measure could guide the development of education and training for this unique provider group.

## 2. Purpose

The main purpose of this study is to evaluate an instrument for use with community pharmacists that assesses knowledge, attitudes, behaviors, and intentions related to IPV and IPV screening. An instrument such as this is necessary for two reasons. First, it allows a standardized evaluation of pharmacists’ knowledge, attitudes, behaviors, and intentions related to IPV and IPV screening that can be compared to other health care providers. Secondly, the data collected with this instrument can guide the development of future educational initiatives, policy recommendations, and potentially the future development of screening programs in the community pharmacy setting. Any differences between the pharmacist version and other versions are important to understand as these potential differences have implications for the design, implementation, and evaluation of interventions to prepare pharmacists to provide high quality care to patients exposed to intimate partner violence.

## 3. Materials and Methods

### 3.1. Study Design

This study utilized a cross-sectional online survey. The study was approved by the University of Mississippi Institutional Review Board (protocol #12-235).

### 3.2. Instrument

This study was designed to adapt and evaluate the PREMIS instrument in pharmacists. Modifications to develop a PREMIS for Pharmacists instrument occurred in several steps. First, the original PREMIS items were adapted for use with pharmacists to address the unique practice characteristics, activities, and concerns of a pharmacy practitioner. Additionally, the opinions section was adapted to reword clinical examination terms and items to assess intention to screen were added as it is anticipated that few pharmacists have conducted screening to date, so assessing screening behavior only is less optimal for this population. Finally, the demographic and IPV history items were adapted as the PREMIS instrument did not use standardized demographic or IPV history items. The demographic and IPV history items were replaced with those utilized in the national Behavioral Risk Factor Surveillance System (BRFSS) surveys. Next, we conducted an expert review of the measure by researchers with expertise in IPV screening, community pharmacy practice, and health behavior theory. Suggested revisions were incorporated and a pilot instrument was finalized. Before the instrument was pilot tested, cognitive interviews were conducted. Cognitive interviews, a recommended step prior to administering a pilot survey, can detect any challenges in understanding navigation, wording of directions and questions, visual layout, etc. [37]. Three cognitive interviews were conducted with a convenience sample of practicing community pharmacists. Revisions based on the cognitive interviews were made and the revised instrument was then pilot tested with a convenience sample of faculty in a school of pharmacy and local practicing community pharmacists. The final survey instrument was then programmed into Qualtrics, an online survey system, for administration with the study sample (see Supplementary Material 1 for the complete survey).

### 3.3. Survey Administration

The adapted measure, PREMIS for Pharmacists, was distributed via email in March 2012 to a national sample of 6000 community pharmacists enrolled in a panel with Integrated Medical Data, a data services company. Multiple email prompts, following the tailored design method, and a $10 gift card incentive were utilized to encourage participation with the survey remaining open for 8 weeks [38]. A total of 189 pharmacists responded. After a review of the data, 45 responses were not included in the analyses as they had not completed 90% of the survey, resulting in a final analytic sample of 144 participants. Once the final analytic sample was prepared, coding was completed, including reverse scoring relevant items.

### 3.4. Psychometric Studies

Maximum likelihood exploratory factor analysis was utilized to evaluate the psychometric properties of the adapted PREMIS for Pharmacists instrument. Results were compared to the original results by Short et al. and to the results of the instrument adapted for use in two additional populations, health care students and Greek physicians [34,35,36]. Exploratory factor analysis was appropriate for this study as the measure being tested has only been utilized in three studies, two of which utilized an adaptation of the original instrument and found slightly different factor structures compared to the original study. The current study adapted the measure for use with practicing community pharmacists and this adapted measure has never been tested before. Because the training and practice of community pharmacists are considerably different from physicians, a different factor structure was possible. All analyses were conducted utilizing SPSS 20.0. Three steps were taken prior to analysis to examine the factorability of the data. First, the variable-to-case ratio was calculated to determine if the study met the recommendation of a 1:5–10 ratio for factor analysis [39]. Second, Bartlett’s test for sphericity was estimated to test for the presence of correlations among the variables. Finally, the Kaiser–Meyer–Olkin measure of sampling adequacy (KMO MSA) was calculated.

Maximum likelihood exploratory factor analysis with an oblique rotation based on eigenvalues greater than one was used to replicate the analysis approach that was used in all three of the studies of this instrument [34,35,36]. This iterative method of factor analysis is a preferred extraction method because it employs a statistical test to determine the number of factors to be extracted. The procedure begins with one factor and adds one factor in each iteration until the model achieves goodness of fit as demonstrated by the *X*^2^ test. Once the appropriate number of factors has been determined, the extracted factors will be subjected to oblique rotation to foster interpretability. Oblique rotation was selected because it is anticipated that the factors may be intercorrelated and oblique rotation allows this, whereas orthogonal rotation does not. Following the recommendation of Thompson, the promax method of oblique rotation was utilized with a pivot power of 4 [40]. Factors were examined for both statistical and theoretical soundness. Items were considered for deletion if a factor loading was lower than 0.32 or if an item cross-loaded on multiple factors. Only factor loadings greater than 0.20 were displayed in the analysis. Reliability and validity were then evaluated.

Validity was examined in several ways, including following Schaffer, DeGeest, and Li’s 2016 guidelines to assess discriminant validity [41]. Construct validity assessment followed Flake, Pek, and Hehman’s 2017 guidance to examine substantive, structural, and external evidence [42]. Substantive evidence included expert review and cognitive interviews; structural evidence included item analysis, factor analysis, and reliability assessments, including Cronbach’s alpha to examine internal consistency within identified scales. Further, correlation between the scales was used in the assessment. Correlations were considered weak if *r* < 0.30, moderate if *r* is between 0.30 and 0.70, and strong if *r* > 0.70. External evidence in the form of comparison with previous administrations of PREMIS in other populations was also conducted. Statistical significance for all tests was set at α < 0.05.

## 4. Results

### 4.1. Study Participants

The mean age of the pharmacists in this study was 47.9 years (±11.8 years), with a range of 28 to 80 years of age. Table 2 reports the sex and race of the study participants. In order to characterize the training characteristics, participants were asked to report their most advanced pharmacy training and to indicate any postgraduate training they may have had. The majority of participants had either a B.S. in Pharmacy or a Pharm.D. Participants also reported completion of training in areas such as anticoagulation therapy, diabetes education, nuclear pharmacy, health care management, and geriatric care. Participants reported that they have been practicing an average of 23.3 years (±12.5) (range 0 to 60), including their residency. Most of the participants had no IPV-related training (67.4%). For those that had any training, they reported their training included reading their institution’s protocol (13.2%), watching a video (11.1%), or attending a lecture on IPV (9.0%). Additional participant characteristics are included in Supplementary Material 2.

### 4.2. Measurement Model Exploration

#### 4.2.1. Background Scales

The original PREMIS instrument had three background scales assessing Perceived Knowledge, Perceived Preparation, and Actual Knowledge and these scales were adapted and utilized in the PREMIS for Pharmacists. The Perceived Preparation scale included 12 items that assessed how prepared pharmacists felt to work with IPV victims and responses ranged from 1 (not prepared) to 7 (quite well prepared). The mean composite score on this 12-item scale was 27.76 (±17.28). The internal consistency of this scale was high (α = 0.970). The Perceived Knowledge scale contained 16 items that assessed respondents’ perceived knowledge about IPV. Responses on these items ranged from 1 (nothing) to 7 (very much) and the mean composite scale score was 35.36 (±23.06). The internal consistency of this scale was also high (α = 0.978). The IPV Actual Knowledge scale included 18 items, with a possible range from 6 to 32, and the mean composite score on this scale was 20.83 (±6.04).

#### 4.2.2. Opinion Scales

Exploratory factor analysis was employed with the 32 opinion items of the PREMIS for Pharmacists instrument to explore and refine the underlying structure of the items in this population. In order to determine the factorability of the data in this sample, the variable-to-case ratio was examined. A total number of 32 variables was considered in this analysis, making the variable-to-case ratio 32 to 144. The Kaiser–Meyer–Olkin (KMO) measure of sampling adequacy was 0.731, indicating the suitability of the data for factor analysis. Bartlett’s test of sphericity was significant (*X*^2^ = 2370.63; df = 465; *p* < 0.001), indicating sufficient correlation between the items and thus the appropriateness of factor analysis for these data. Maximum Likelihood Factor (MLF) analysis with oblique rotation of the 32 opinion items identified a 9-factor solution that was statistically sound (*X*^2^ = 277.57; df = 222; *p* < 0.007) that explained 54.65% of the variance; however, 23 of the items had similar loadings in at least two factors, indicating complex loadings. Although this solution was statistically sound, the solution lacked a good theoretical basis. Variables with low communalities or loading scores below 0.32 were removed from analysis. The final MLF factor solution had five factors utilizing 18 items and accounted for 64.16% of the variance. Only loadings greater than 0.20 were shown; all of the items loaded exclusively on one factor in the final solution. Four of the five identified scales had Cronbach’s α > 0.70 and were thus considered to have acceptable reliability. The fifth scale demonstrated moderate reliability (α = 0.676). The identified scales, with reliability coefficients and sample items, are included in Table 3.

#### 4.2.3. Assessing Validity

Utilizing the guidance of Shaffer, DeGeest, and Li (2016) and Flake, Pek, and Hehman (2017), validity was assessed in multiple steps starting with a literature review to look for any similar measures in the field of pharmacy [41]. As none were found, the next step was to evaluate construct validity utilizing a multitrait, multimethod matrix. This was assessed by estimating the correlation between the instrument’s scales. Similar to both Short et al. and Connor et al., in the PREMIS for Pharmacists, the Perceived Knowledge score was strongly correlated with the Perceived Preparation score (*r* = 0.889; *p* = 0.01) and moderately correlated with the amount of previous training (*r* = 0.402; *p* = 0.01); a moderate correlation between Perceived Preparation and hours of previous IPV training was also found (*r* = 0.402; *p* = 0.01). Similar to Connor et al., we found no significant correlation between Actual Knowledge of IPV and Perceived Knowledge, Perceived Preparation, and hours of previous IPV training (Table 4).

In contrast, Short et al. did find a correlation between Actual Knowledge and Perceived Knowledge (*r =* 0.201). Perceived Preparation and Perceived Knowledge were significantly correlated with all of the Opinion scales, although none of the correlations were strong (Table 5). These findings are also similar to Short et al. and Connor et al. Finally, the Opinion scales were examined and a moderate correlation between Workplace and Self-Efficacy and Legal Requirements was found (*r* = 0.526; *p* < 0.01). Weak but significant correlations were found among several of the other Opinion scales (Table 6).

#### 4.2.4. Comparison with Other PREMIS Versions

The PREMIS for Pharmacists was compared to the factor structure of PREMIS previously identified in samples of physicians and health care students. The background scales of Perceived Preparation and Perceived Knowledge function similarly in the pharmacist sample as they did in all three of the previous studies. The same factor analytic strategy of the items related to opinions of IPV and IPV screening that was used in all three of the previous studies was utilized in this study. Several of the same factors were identified (Preparation, Legal Requirements, Alcohol and Drugs, Constraints). While the Opinions scale did find several of the same factors, there were a number of differences between the PREMIS for Pharmacists and the other studies. First, the PREMIS for Pharmacists identified a single factor for self-efficacy and workplace-efficacy, whereas the previous studies found these to be two separate factors. Second, the number of items in some of the scales was not identical. For example, the preparation scale had fewer items in the PREMIS for Pharmacists (3 items) compared to the physician PREMIS (5 items) and the student and Greek physician versions (4 items each). Finally, several of the factors identified in the original instrument (victim understanding and victim autonomy) and in the other adaptations of the instrument (IPV screening) were not found in the PREMIS for Pharmacists.

## 5. Discussion

The PREMIS for Pharmacists instrument was adapted and evaluated in a national random sample of practicing community pharmacists. This new measure, the PREMIS for Pharmacists, was found to be a valid tool that can be used with pharmacists to assess baseline knowledge, attitudes, behaviors, and intentions regarding IPV-related care. This measure would therefore provide a valid method to assess the potential impact of education and training programs related to IPV.

Importantly, a similar, but not identical, factor structure was found in the PREMIS for Pharmacists compared to previous studies with PREMIS in other, nonpharmacist populations. The background scales, including Perceived Preparation, Perceived Knowledge, and Actual Knowledge, translated well to the pharmacy setting. The factor structure of the opinions component of the instrument found four of the original factors (Preparation, Legal Requirements, Alcohol and Drugs, and Constraints). However, the pharmacy version found a single factor, which we labeled Workplace and Self-Efficacy, that was split into two separate factors (Workplace Issues and Self-Efficacy) in the previous studies. One reason for this finding may be related to the self-reported level of training and clinical experience with IPV and IPV screening. The pharmacists reported less training and experience compared to the other health care provider populations. The lack of knowledge and awareness of the details of the challenges related to IPV screening may have made it difficult for pharmacists to tease apart the efficacy issues related to themselves as clinicians as compared to their work environments. If educational and training initiatives for pharmacists increase, this may change and the factor structure should be re-evaluated. It is also interesting to note that the Victim Understanding and Victim Autonomy scales were not found in the PREMIS for Pharmacists. Both of these scales had low reliability in the previous studies and it was recommended that they be further explored. This is another example of how the lack of pharmacists’ training and exposure to IPV screening recommendations and IPV screening programs may have impacted this finding. These results indicate that pharmacists do not have well-formed clinical opinions regarding IPV victims in general and educational and training initiatives may impact this.

There are several limitations to the current study. First, this survey covered a sensitive topic and this may have reduced response rate and may also have resulted in individuals responding with what they perceived to be more socially desirable responses. Second, the response rate to this survey was low, which may impact the generalizability of the results. Further, this was a cross-sectional survey study and as such, test–retest reliability could not be evaluated. Given that this was an online survey study, only participants with internet access could participate. Selective participation is an additional risk that could bias the results. The sample was drawn from a panel of pharmacists, further limiting the generalizability of the results.

Future studies are needed to further test the PREMIS for Pharmacists, especially test–retest reliability assessments, assessment in other pharmacy populations (e.g., pharmacy students, hospital pharmacists), as well as investigation of whether the instrument is able to detect improvement after IPV-related training. Additionally, given that pharmacists are the most accessible members of the health care team from both a cost and access perspective, the potential to expand screening to the pharmacy environment warrants further study. This new tool could help identify pharmacists’ readiness to detect and support patients who may be experiencing IPV. In this manner, pharmacists, like other health care practitioners, could play a crucial frontline role in helping to identify individuals exposed to IPV and providing assistance and referrals.

## 6. Conclusions

Intimate partner violence is highly prevalent and has a negative impact on health and health behaviors, including medication adherence. Pharmacists receive minimal training regarding IPV and no instrument has been available to assess their readiness to care for these patients or the impact of potential IPV educational initiatives. This study demonstrated that the PREMIS for Pharmacists could fill this void. As with the original PREMIS and the Health Care Student PREMIS, the PREMIS for Pharmacists can be utilized in a variety of ways. The instrument can be used to conduct needs assessments to tailor education and training initiatives for pharmacists, as a pre- and posttest to evaluate the impact of training, and to identify differences between pharmacists who have participated in training and those who have not. The PREMIS for Pharmacists provides a key component in efforts to engage pharmacists in addressing the public health challenge of intimate partner violence.

## Figures and Tables

**Table 1:** PREMIS Scales in Prior Studies.

	Short et al. Physicians *n* = 67			Connor et al. Health Professions Students *n* = 286			Papadaki et al. Greek Physicians *n* = 80		
Scales	Alpha	Total Items	Mean (SD) ^1^	Alpha	Total Items	Mean (SD) ^1^	Alpha	Total Items	Mean (SD) ^1^
BACKGROUND									
Perceived Preparation	0.96	12	3.67 (1.05)	0.97	12	3.80 (1.52)	0.93	9	4.08 (1.17)
Perceived Knowledge	0.96	16	3.55 (0.97)	0.97	16	3.83 (1.42)	0.96	16	3.36 (1.22)
Actual Knowledge	n/a	18	26.0 (5.18)	n/a	18	23.9 (5.68)	n/a	18	18.52 (4.58)
OPINIONS									
Preparation	0.85	5	4.20 (1.11)	0.89	4	NR ^2^	0.78	4	3.70 (1.24)
Legal Requirements	0.82	4	3.92 (1.15)	0.91	3	NR	−	−	−
Workplace Issues	0.79	6	4.18 (1.05)	−	−	−	0.78	5	3.09 (1.13)
Self-efficacy	0.69	6	3.68 (1.26)	0.80	7	NR	0.75	3	4.78 (1.22)
Alcohol & Drugs	0.70	3	4.46 (0.61)	0.48	2	NR	0.50	2	4.05 (0.80)
Victim Understanding	0.69	7	5.06 (0.78)	0.46	3	NR	0.63	4	4.10 (1.24)
Constraints	0.47	2	4.65 (1.26)	−	−	−	0.61	3	4.33 (1.38)
Victim Autonomy	0.37	3	4.32 (0.83)	0.36	3	NR	−	−	−
IPV Screening	−	−	−	0.74	2	NR	0.58	2	34.45 (1.40)

^1^ On a scale from 1 (strongly disagree) to 7 (strongly agree); ^2^ NR = not reported.

**Table 2:** Participant Demographics.

	Percent	*n*
Sex		
Female	52.8%	76
Male	47.2%	68
Race		
White	84.7%	122
Black/African American	3.5%	5
Asian	7.6%	11
Native Hawaiian/Pacific Islander	0.7%	1
American Indian/Alaskan Native	0.7%	1
Other	2.8%	4
Hispanic		
Yes	5.6%	8
No	93.8%	135
Don’t know/Not sure	0.7%	1
Most advanced pharmacy training		
B.S. in Pharmacy	59.7%	86
Pharm.D.	37.5%	54
M.S. in Pharmacy	2.1%	3
Other	0.7%	1

**Table 3:** PREMIS for Pharmacists Scales.

Scales	Alpha	Total Items	Item Mean (SD) ^1^	Sample Item
BACKGROUND				
Perceived Preparation	0.97	12	2.31 (0.003)	How prepared do you feel to appropriately respond to disclosure?
Perceived Knowledge	0.98	16	2.21 (0.004)	How much do you feel you know about what questions to ask to identify IPV?
Actual Knowledge	n/a	18	20.83 (6.04)	What is the strongest single risk factor for being a victim of IPV?
Practice Issues	n/a	21	9.44 (6.95)	For every IPV victim you have identified in the past 6 months, how often have you documented patient’s statements about IPV in record?
OPINIONS				
Efficacy-workplace/self	0.86	7	2.68 (0.013)	I feel comfortable discussing IPV with my patients. My practice setting allows me adequate time to respond to victims of IPV.
Preparation	0.96	3	3.01 (0.0001)	I don’t have the necessary skills to discuss abuse with an IPV victim who is female.
Legal Requirements	0.95	3	2.93 (0.007)	I am aware of the legal requirements in this state regarding reporting of suspected case of IPV.
Alcohol & Drugs	0.80	2	4.63 (0.01)	Use of alcohol or drugs is related to IPV victimization.
Constraints	0.68	3	4.31 (0.031)	Pharmacists do not have the time to assist patients in addressing IPV.

^1^ On a scale from 1 (strongly disagree) to 7 (strongly agree).

**Table 4:** Correlation between Preparation and Knowledge Items/Scales in PREMIS for Pharmacists.

	Perceived Preparation	Perceived Knowledge	Actual Knowledge	Practice Issues	Hours IPV Training
Perceived Preparation	1				
Perceived Knowledge	0.889 **	1			
Actual Knowledge	0.119	0.106	1		
Practice Issues	0.126	0.086	−0.041	1	
Hours IPV Training	0.409 **	0.402 **	0.213	0.126	1

** *p* < 0.01 (all two-tailed).

**Table 5:** Correlation between Opinion and Background Scale Items/Scales in PREMIS for Pharmacists.

	Perceived Preparation	Perceived Knowledge	Actual Knowledge	Hours IPV Training
Workplace/Self-Efficacy	0.606 **	0.623 **	0.129	0.323 *
Staff Preparation	0.262 **	0.243 **	0.009	0.268
Legal Requirements	0.531 **	0.636 **	0.240 **	0.084
Alcohol/Drugs	0.277 **	0.245 **	−0.110	0.174
Constraints	−0.022	−0.052	0.175	0.309 *

* *p* < 0.05; ** *p* < 0.01 (all two-tailed).

**Table 6:** Correlation between Opinion Scales in PREMIS for Pharmacists.

	Work/Self-Efficacy	Staff Preparation	Legal Requirements	Alcohol/Drugs	Constraints
Work/Self-Efficacy	1				
Staff Preparation	0.217 **	1			
Legal Requirements	0.526 **	0.155	1		
Alcohol/Drugs	0.241 **	−0.074	0.168 *	1	
Constraints	0.125	0.174 *	−0.238 **	−0.342	1

* *p* < 0.05; ** *p* < 0.01 (all two-tailed).

## Supplementary Materials

The following are available online at http://www.mdpi.com/2226-4787/6/3/66/s1, Pharmacist PREMIS Instrument.
