# Supplementary material for: Evaluation of a Tool to Measure Pharmacists’ Readiness to Manage Intimate Partner Violence
Source: Pharmacy (Basel). 2018 Jul 12;6(3):66. doi: 10.3390/pharmacy6030066 (PMC6163905; doi:10.3390/pharmacy6030066)
Supplement: Supplementary file 1 [file pharmacy-06-00066-s001.zip › pharmacy-299360-supplementary-2.pdf]

Supplement 2 Additional Participant Characteristics

**Training Characteristics**	**Percent**	**n**
Postgraduate training		
Residency	16.0%	23
Fellowship	2.8%	4
Graduate School	19.4%	28
Other	31.9%	46
**Practice Characteristics**	**Percent**	**n**
Type of pharmacy		
Chain	11.8%	17
Grocery/General Merchandise	28.5%	41
Independent	34.0%	49
Other	32.6%	47
Position		
Owner/Partner	18.1%	26
Employee manager/Assistant Manager	27.8%	40
Staff/employee pharmacist	47.2%	68
Relief pharmacist	2.1%	3
Other	4.9%	7
Extent involved in key decisions		
Not at all	15.3%	22
A little	17.4%	25
Some	16.0%	23
A good amount	27.1%	39
To a great extent	24.3%	35
Offer advanced pharmacy services		
YES	53.5%	77
NO	46.5%	67
Have a dedicated private counseling area		
YES	57.6%	83
NO	42.4%	61
Frequency of use of private counseling area		
Never	--	0
Rarely	13.3%	11
Sometimes	51.8%	43
Often	34.9%	29
	**Mean (±SD)**	
Average number of fills per day in your pharmacy	326.3 (±674.3)	
Percent of fills involving patient counseling other than ordinary prescription consultation	22.7% (±26.3)	
Average number of pharmacist FTEs in your pharmacy	5.76 (±17.1)	
Average number of pharmacy tech FTEs in your pharmacy	7.58 (±26.8)	

Geographic Distribution of Participants by State	% reported residing in each state
Alabama	1.01%
Alaska	0.00%
Arizona	1.52%
Arkansas	1.52%
California	4.55%
Colorado	2.53%
Connecticut	0.00%
Delaware	1.01%
District of Columbia	0.00%
Florida	4.04%
Georgia	1.52%
Hawaii	1.01%
Idaho	0.00%
Illinois	4.04%
Indiana	3.54%
Iowa	1.01%
Kansas	2.02%
Kentucky	1.01%
Louisiana	0.51%
Maine	2.02%
Maryland	2.53%
Massachusetts	1.52%
Michigan	5.05%
Minnesota	3.03%
Mississippi	0.00%
Missouri	4.04%
Montana	0.51%

Nebraska	1.01%
Nevada	1.01%
New Hampshire	1.52%
New Jersey	3.03%
New Mexico	0.51%
New York	6.57%
North Carolina	5.56%
North Dakota	1.01%
Ohio	6.06%
Oklahoma	0.00%
Oregon	0.00%
Pennsylvania	7.07%
Puerto Rico	0.00%
Rhode Island	0.00%
South Carolina	1.01%
South Dakota	0.00%
Tennessee	2.53%
Texas	3.54%
Utah	0.51%
Vermont	0.51%
Virginia	2.02%
Washington	3.54%
West Virginia	0.00%
Wisconsin	3.54%
Wyoming	0.00%
