# Supplementary material for: Evaluation of a Tool to Measure Pharmacists’ Readiness to Manage Intimate Partner Violence
Source: Pharmacy (Basel). 2018 Jul 12;6(3):66. doi: 10.3390/pharmacy6030066 (PMC6163905; doi:10.3390/pharmacy6030066)
Supplement: Supplementary file 1 [file pharmacy-06-00066-s001.zip › Pharmacy_supplementary 1.pdf]

**Supplement 1**

# Pharmacy PREMIS

Your candid responses on the following survey will greatly assist us in our attempt to improve pharmacists' recognition and management of intimate partner violence-related injuries and illnesses. Please record your first, instinctive answer, even if you don't think it is "politically correct." Don't try to think about what your answers "should" be. Some questions may seem similar to others. However, we ask that you answer all the questions to help ensure the reliability of the assessment. Thank you for taking the time (estimated at 20 minutes) to complete this survey. (Intimate partner violence (IPV) is also commonly referred to as domestic violence (DV), partner violence, or family violence. It is typically between intimate partners including spouses or boy/girlfriends.)

What is your current age?

________________________________________________________________

Sex

○ Male (1)

○ Female (2)

Are you Hispanic or Latino?

○ Yes (1)

○ No (2)

○ Don't know (3)

Which one or more of the following would you say is your race?

▢ White (1)

▢ Black or African American (2)

▢ Asian (3)

▢ Native Hawaiian or other Pacific Islander (4)

▢ American Indian or Alaskan Native (5)

▢ Other (6)

In which state is the pharmacy in which you work located? (*If you work in more than one pharmacy, indicate the state for the pharmacy you consider to be your primary location.)*

▼ Alabama (1) ... I do not reside in the United States (53)

Which one of the following best describes your **most advanced** pharmacy training? (Please check only one)

○ B.S. in Pharmacy (1)

○ Pharm.D. (2)

○ M.S. in Pharmacy (3)

○ Other (please specify): (4) ____________________

In which year did you graduate with your last professional degree?

____________________

Which type of postgraduate training have you completed? (Please check ALL that apply)

☐ Residency (1)

☐ Fellowship (2)

☐ Graduate School (3)

☐ Other (please specify): (4) ____________________

Including your residency, how many years have you been actively practicing pharmacy? (Please round to the nearest whole year)

____________________

What type of pharmacy do you currently work in?  (Please check ALL that apply)

- ☐ Chain pharmacy (e.g., Walgreens, CVS)  (1)
- ☐ Pharmacy in grocery or general merchandise store (e.g., Kroger, Walmart, Fred's, etc.)  (2)
- ☐ Independent pharmacy  (3)
- ☐ Other (please specify):  (4) ________________________________________________

Including yourself, how many practitioners (pharmacists and/or pharmacy technicians) at your work site have participated in an intimate partner violence or domestic violence training course in the past 6 months?

- ○ All  (1)
- ○ Most  (2)
- ○ Some  (3)
- ○ A few  (4)
- ○ None  (5)
- ○ Don't know  (6)

How much previous training about intimate partner violence issues have you had? *(Please mark all that apply.)*

- ☐ None (1)
- ☐ Read my institution's protocol (2)
- ☐ Watched a video (3)
- ☐ Attended a lecture or a talk (4)
- ☐ Attended a skills-based training or workshop (5)
- ☐ Pharmacy/other school classroom workshop (6)
- ☐ Pharmacy/other school clinical training (7)
- ☐ Residency/fellowship/post graduate training (8)
- ☐ Continuing Education (9)
- ☐ Other in-depth training (more than 4 hours) (10)
- ☐ Other (please specify): (11) ________________________________________

Estimated total number of hours of previous intimate partner violence training:

________________________________________

Page Break

PERCEIVED PREPARATION

Please indicate which best describes how prepared you feel to perform the following:

| | Not prepared 1 (1) | 2 (2) | 3 (3) | 4 (4) | 5 (5) | 6 (6) | Quite well prepared 7 (7) |
|---|---|---|---|---|---|---|---|
| Ask appropriate questions about intimate partner violence (IPV) (1) | ○ | ○ | ○ | ○ | ○ | ○ | ○ |
| Appropriately respond to disclosures of abuse (2) | ○ | ○ | ○ | ○ | ○ | ○ | ○ |
| Identify IPV indicators based on patient's history (3) | ○ | ○ | ○ | ○ | ○ | ○ | ○ |
| Assess an IPV victim's readiness to change (4) | ○ | ○ | ○ | ○ | ○ | ○ | ○ |
| Help an IPV victim assess his/her danger of lethality (5) | ○ | ○ | ○ | ○ | ○ | ○ | ○ |
| Conduct a safety assessment for the victim's children (6) | ○ | ○ | ○ | ○ | ○ | ○ | ○ |
| Help an IPV victim create a safety plan (7) | ○ | ○ | ○ | ○ | ○ | ○ | ○ |
| Document IPV history findings in patient's record (8) | ○ | ○ | ○ | ○ | ○ | ○ | ○ |
| Make appropriate referrals for IPV (9) | ○ | ○ | ○ | ○ | ○ | ○ | ○ |
| Fulfill reporting requirements for IPV (10) | ○ | ○ | ○ | ○ | ○ | ○ | ○ |
| Fulfill reporting requirements for child abuse (11) | ○ | ○ | ○ | ○ | ○ | ○ | ○ |
| Fulfill reporting requirements for elder abuse (12) | ○ | ○ | ○ | ○ | ○ | ○ | ○ |

PERCEIVED KNOWLEDGE
How much do you feel you know about:

| | Nothing 1 | 2 (2) | 3 (3) | 4 (4) | 5 (5) | 6 (6) | Very much 7 |
|---|---|---|---|---|---|---|---|
| Your legal reporting requirements for Intimate Partner Violence (IPV) (1) | ○ | ○ | ○ | ○ | ○ | ○ | ○ |
| Your legal reporting requirements for child abuse (2) | ○ | ○ | ○ | ○ | ○ | ○ | ○ |
| Your legal reporting requirements for elder abuse (3) | ○ | ○ | ○ | ○ | ○ | ○ | ○ |
| Signs or symptoms of IPV (4) | ○ | ○ | ○ | ○ | ○ | ○ | ○ |
| How to document IPV in patient's record (5) | ○ | ○ | ○ | ○ | ○ | ○ | ○ |
| Referral sources for IPV victims (6) | ○ | ○ | ○ | ○ | ○ | ○ | ○ |
| Perpetrators of IPV (7) | ○ | ○ | ○ | ○ | ○ | ○ | ○ |
| Relationship between IPV and pregnancy (8) | ○ | ○ | ○ | ○ | ○ | ○ | ○ |
| Recognize the childhood effects of witnessing IPV (9) | ○ | ○ | ○ | ○ | ○ | ○ | ○ |
| What questions to ask to identify IPV (10) | ○ | ○ | ○ | ○ | ○ | ○ | ○ |
| Why a victim might not disclose IPV (11) | ○ | ○ | ○ | ○ | ○ | ○ | ○ |
| Your role in detecting IPV (12) | ○ | ○ | ○ | ○ | ○ | ○ | ○ |
| What to say and not say in IPV situations with a patient (13) | ○ | ○ | ○ | ○ | ○ | ○ | ○ |

| | | | | | | | |
|---|---|---|---|---|---|---|---|
| Determining danger for a patient experiencing IPV (14) | ○ | ○ | ○ | ○ | ○ | ○ | ○ |
| Developing a safety plan with an IPV victim (15) | ○ | ○ | ○ | ○ | ○ | ○ | ○ |
| The stages an IPV victim experiences in understanding and changing her/his situation (16) | ○ | ○ | ○ | ○ | ○ | ○ | ○ |

IPV KNOWLEDGE
Check one answer per item, unless noted otherwise.

What is the strongest *single* risk factor for becoming a victim of intimate partner violence?

- ○ Age (  (1)
- ○ Partner abuses alcohol/drugs  (2)
- ○ Gender - female  (3)
- ○ Family history of abuse  (4)
- ○ Don't know  (5)

Which *one* of the following is generally true about batterers?

- ○ They have trouble controlling their anger  (1)
- ○ They use violence as a means of controlling their partners  (2)
- ○ They are violent because they drink or use drugs  (3)
- ○ They pick fights with anyone  (4)

Which of the following are warning signs that a patient may have been abused by his/her partner? *(check all that apply)*

- ☐ Chronic unexplained pain (1)
- ☐ Anxiety (2)
- ☐ Substance abuse (3)
- ☐ Frequent injuries (4)
- ☐ Depression (5)

Which of the following are reasons an IPV victims may not be able to leave a violent relationship? (*check all that apply*)

- ☐ Fear of retribution (1)
- ☐ Financial dependence on the perpetrator (2)
- ☐ Religious beliefs (3)
- ☐ Children's needs (4)
- ☐ Love for one's partner (5)
- ☐ Isolation (6)

---

Which of the following is/are the most appropriate ways to ask about IPV? (check all that apply)

- ☐ "Are you a victim of intimate partner violence?" (1)
- ☐ "Has your partner ever hurt or threatened you?" (2)
- ☐ "Have you ever been afraid of your partner?" (3)
- ☐ "Has your partner ever hit or hurt you?" (4)

Which of the following is/are generally true? *(check all that apply)*

- ☐ There are common non-injury presentations in abused patients (1)
- ☐ There are behavioral patterns in couples that may indicate IPV (2)
- ☐ Specific areas of the body are most often targeted in IPV cases (3)
- ☐ There are common injury patterns associated with IPV (4)
- ☐ Injuries in different stages of recovery may indicate abuse (5)

Mark TRUE, FALSE, or DON'T KNOW the answer to the following questions:

Alcohol consumption is the greatest single predictor of the likelihood of Intimate Partner Violence (IPV).

- TRUE (1)
- FALSE (2)
- Don't know (3)

There are good reasons for not leaving an abusive relationship.

- TRUE (1)
- FALSE (2)
- Don't know (3)

Reasons for concern about Intimate Partner Violence (IPV) should not be included in a patient's record if s/he does not disclose the violence.

- TRUE (1)
- FALSE (2)
- Don't know (3)

When asking patients about Intimate Partner Violence (IPV), providers should use the words "abused" or "battered".

- TRUE (1)
- FALSE (2)
- Don't know (3)

Being supportive of a patient's choice to remain in a violent relationship would condone the abuse.

◯ TRUE (1)

◯ FALSE (2)

◯ Don't know (3)

Victims of Intimate Partner Violence (IPV) are able to make appropriate choices about how to handle their situation.

◯ TRUE (1)

◯ FALSE (2)

◯ Don't know (3)

Health care providers should not pressure patients to acknowledge that they are living in an abusive relationship.

◯ TRUE (1)

◯ FALSE (2)

◯ Don't know (3)

Victims of Intimate Partner Violence (IPV) are at greater risk of injury when they leave the relationship.

◯ TRUE (1)

◯ FALSE (2)

◯ Don't know (3)

Strangulation injuries are rare in cases of Intimate Partner Violence (IPV).

- TRUE (1)
- FALSE (2)
- Don't know (3)

Allowing partners or friends to be present during a patient's history and physical exam ensures safety for an IPV victim.

- TRUE (1)
- FALSE (2)
- Don't know (3)

Even if the child is not in immediate danger, pharmacists in all states are mandated to report an instance of a child witnessing IPV to Child Protective Services.

- TRUE (1)
- FALSE (2)
- Don't know (3)

OPINIONS

Please indicate your level of agreement with each of these statements about Intimate Partner Violence (IPV):

| | Strongly Disagree 1 | 2 | 3 | 4 | 5 | 6 | Strongly Agree 7 |
|---|---|---|---|---|---|---|---|
| I feel comfortable discussing IPV with my patients. | ○ | ○ | ○ | ○ | ○ | ○ | ○ |
| I don't have the necessary skills to discuss abuse with an IPV victim who is female. | ○ | ○ | ○ | ○ | ○ | ○ | ○ |
| I don't have the necessary skills to discuss abuse with an IPV victim who is male. | ○ | ○ | ○ | ○ | ○ | ○ | ○ |
| I don't have the necessary skills to discuss abuse with an IPV victim who is from a different cultural/ethnic background. | ○ | ○ | ○ | ○ | ○ | ○ | ○ |
| I am aware of legal requirements in this state regarding reporting of suspected cases of IPV. | ○ | ○ | ○ | ○ | ○ | ○ | ○ |
| I am aware of legal requirements in this state regarding reporting of suspected cases of child abuse. | ○ | ○ | ○ | ○ | ○ | ○ | ○ |
| I am aware of legal requirements in this state regarding reporting of suspected cases of elder abuse. | ○ | ○ | ○ | ○ | ○ | ○ | ○ |

Please indicate your level of agreement with each of these statements about Intimate Partner Violence (IPV):

| | Strongly Disagree 1 | 2 | 3 | 4 | 5 | 6 | Strongly Agree 7 |
|---|---|---|---|---|---|---|---|
| Pharmacists do not have the time to assist patients in addressing IPV. | ○ | ○ | ○ | ○ | ○ | ○ | ○ |
| I am able to gather the necessary information to identify IPV as the underlying cause of patient illness (e.g. depression, migraines). | ○ | ○ | ○ | ○ | ○ | ○ | ○ |
| Pharmacists have a responsibility to ask all patients about IPV. | ○ | ○ | ○ | ○ | ○ | ○ | ○ |
| My practice setting allows me adequate time to respond to victims of IPV. | ○ | ○ | ○ | ○ | ○ | ○ | ○ |
| I have contacted services within the community to establish referrals for IPV victims. | ○ | ○ | ○ | ○ | ○ | ○ | ○ |
| Alcohol abuse is a leading cause of IPV. | ○ | ○ | ○ | ○ | ○ | ○ | ○ |
| I am too busy to participate on a multidisciplinary team that manages IPV cases. | ○ | ○ | ○ | ○ | ○ | ○ | ○ |

Please indicate your level of agreement with each of these statements about Intimate Partner Violence (IPV):

| | Strongly Disagree 1 | 2 | 3 | 4 | 5 | 6 | Strongly Agree 7 |
|---|---|---|---|---|---|---|---|
| Screening for IPV is likely to offend those who are screened. | ○ | ○ | ○ | ○ | ○ | ○ | ○ |
| There is adequate private space for me to provide care for victims of IPV. | ○ | ○ | ○ | ○ | ○ | ○ | ○ |
| I am able to gather the necessary information to identify IPV as the underlying cause of patient medication utilization. | ○ | ○ | ○ | ○ | ○ | ○ | ○ |
| Use of alcohol or other drugs is related to IPV victimization. | ○ | ○ | ○ | ○ | ○ | ○ | ○ |
